# Patterns, Trajectories, and Predictors of Functional Decline after Hospitalization for Acute Exacerbations in Men with Moderate to Severe Chronic Obstructive Pulmonary Disease: A Longitudinal Study

**DOI:** 10.1371/journal.pone.0157377

**Published:** 2016-06-14

**Authors:** Francesc Medina-Mirapeix, Roberto Bernabeu-Mora, Gloria García-Guillamón, Elisa Valera Novella, Mariano Gacto-Sánchez, José Antonio García-Vidal

**Affiliations:** 1 Department of Physical Therapy, Universidad of Murcia, Murcia, Spain; 2 Division of Pneumology, Hospital Morales Meseguer, Murcia, Spain; 3 Department of Physical Therapy, EUSES University of Girona, Girona, Spain; University of Athens, GREECE

## Abstract

**Background:**

Hospitalization for acute exacerbations (AE) of chronic obstructive pulmonary disease (COPD) is common, but little is known about the impact of hospitalization on the development of disability. The purpose of this study was to determine the rate and time course of functional changes 3 months after hospital discharge for AE-COPD compared with baseline levels 2 weeks before admission, and to identify predictors of functional decline.

**Methods:**

This was a prospective study including 103 patients (age mean, 71 years; standard deviation, 9.1 years) who were hospitalized with AE-COPD. Number of dependencies in Activities of Daily Living (ADLs) was measured at the preadmission baseline and at weeks 6 and 12 after discharge. Patterns of improvement, no change, and decline were defined over 3 consecutive intervals (baseline and weeks 6 and 12). Trajectories grouped patients with similar time courses of disability. Recovery was defined as returning to baseline function after functional decline. Univariate and multivariate multiple logistic regression was used to determine predictors of functional decline after week 12.

**Results:**

Six trajectories of functional changes were found. From baseline to 12 weeks, 50% of patients continued to have the same function whereas 31% experienced functional decline after 6 weeks; 16.7% recovered over subsequent weeks. At week 12, as a consequence of all trajectories, 38% of patients showed functional declines compared with baseline function, 57% had not declined, and 6 improved. Length of stay (odds ratio [OR] = 1.12;95% [confidence interval] CI 1.03–1.22), dyspnea (OR = 1.85; 95% CI 1.05–3.26), and frailty (OR = 3.97; 95% CI 1.13–13.92) were independent predictors of functional decline after 12 weeks.

**Conclusions:**

Hospitalization for AE-COPD is a risk factor for the progression of disability. More than one third of patients hospitalized for AE-COPD declined during the 12 weeks following discharge, with most of this decline occurring by week 6.

## Introduction

Patients with chronic obstructive pulmonary disease (COPD) experience greater disability in activities of daily living (ADL) than the general population and those with other chronic health conditions [1]. Disability in ADL often leads to serious short-term consequences for patients and their families since the individuals cannot live at home without assistance from caregivers [2]. Although prior research indicates that COPD-related disability is a substantive problem, very little is known about how the disease progresses to disability [3]. Consequently, there is a need to identify risk factors for the development of disability.

Hospitalization has been shown to increase the risk of functional decline and disability in other populations [4]. Hospitalization for acute exacerbations (AE) of COPD is common in Europe [5]. In some countries, these hospitalizations account for about 10% of all acute medical admissions [5,6]. Despite the high incidence of hospitalization for AE-COPD, relatively little is known about the impact of hospitalization on the development of disability after hospital discharge. Most previous studies on COPD-related disability have been performed in stable outpatients [1,3]. Studies of hospitalized COPD patients have emphasized other outcomes (mortality, readmission rates, lung function measurements, quality of life, or patient satisfaction) [6]. Only one study reported on the effect of hospitalization on disability following discharge [2]. This study reported that, at middle term, more than half of adults hospitalized for AE-COPD subsequently required assistance to perform ADLs. However, dependence measurements were not performed at baseline and it is possible that dependence scores were already present at the beginning of the study. Additionally, the magnitude and determinants of the impact of hospitalization for AE-COPD could not be investigated in the absence of a baseline disability measure [2].

The purpose of this study was to determine the rate and time course of functional changes 3 months after discharge from the hospital for patients experiencing AE-COPD compared with their preadmission baseline rates 2 weeks prior to admission, and to identify predictors of functional decline.

## Materials and Methods

### Study design and participants

A prospective observational design was used. Patients hospitalized with exacerbation of their COPD were prospectively recruited from a hospital at Meseguer Hospital, Murcia, Spain. Outcomes were assessed by face-to-face interviews with patients or surrogate respondents at baseline (T0) and by phone interviews 6 (T1) and 12 (T2) weeks after hospital discharge. Surrogates, who were identified as the primary caregivers, were interviewed when patients were unable to be interviewed or contacted. All study participants provided written informed consent and the study protocol was approved by the institutional review board of the hospital called “Comité Ético de Investigación Clínica del Hospital General Universitario José María Morales Meseguer” (EST-35/13).

Inclusion criteria were a diagnosis of COPD exacerbation upon hospital admission and an age of 40 years or older. The diagnosis of COPD and the stage and severity of the disease were based on the Global Initiative for Chronic Obstructive Lung Disease (GOLD) guidelines [7].

Potential participants were excluded based on following criteria: significant cognitive deficits (i.e., Mini-mental State Examination score of <20), a terminal illness (expected survival of <4 months), living outside of the hospital’s catchment area, a length of stay of >30 days, and not having any control visit for COPD within the 6 months before the hospital admission. Based on patient health examinations and review of patients’ electronic files, a pulmonary physician identified and recruited a consecutive sample of eligible patients during a one-year period.

### Measurements

#### Outcome measure

We used a dependence scale that included 6 ADLs (toileting, bathing, transferring, eating, dressing, and walking across a small room) as described elsewhere [4]. Dependence was defined as a self-report of being unable to perform an ADL or requiring the help of another person for any ADL. The score range of this scale (0–6) was based on the number of dependencies, with a score of 6 representing dependencies in all ADLs. The ADL dependence scale was administered after hospital admission and each patient was asked whether he/she had been able to perform each of 6 ADLs independently 2 weeks before admission and at follow up interviews. This definition for baseline function was chosen because it generally reflects function before the exacerbation of chronic illness resulting in hospital admission, but is recent enough that the patient’s and surrogate’s recall of functional status is reliable [8]. The scale was also administered at follow up interviews, and change scores were calculated.

#### Predictor variables

A total of 12 variables were selected from the literature based on their potential association with functional decline or recovery after hospitalization. These variables were classified into 3 domains; sociodemographic, illness and physiological factors, and hospitalization characteristics. The sociodemographic domain included self-reported age (years), sex, and education level (no studies, primary, and secondary). Illness and physiological factors were related to health status before hospitalization and included forced expiratory volume (FEV_1_), severity of airflow limitation, modified Medical Research Council (mMRC) dyspnea scale, number of medications or prescription drugs used, number of comorbidities measured using the Functional Comorbidity Index [9], and frailty. All these factors except frailty were acquired from electronic files. Frailty was measured after hospital admission by means of the Reported Edmonton Frail Scale, which is based on a scale from 0 to 18, with higher scores entailing more severe frailty [10]. The hospitalization domain included length of stay (days) and number of hospitalizations due to AE in the previous year. The number of hospitalizations was ranked as follows: having 0 to 1 hospitalizations, or having 2 or more hospitalizations. Thus, the patients with two or more hospitalizations were considered as patients with severe AE.

### Statistical analysis

Change scores on the ADL dependence scale were categorized into 3 patterns: decline, no change, or improvement. A difference less than the minimal important difference (MID) between baseline dependence and any specific interval (6 or 12 weeks) was categorized as no change rather than as improvement (positive difference) or decline (negative difference). The MID is the smallest change in a health-related outcome measure that is large enough to trigger a change in management [11]. As described elsewhere [12], we used the 0.5 standard deviation of scores at baseline as a measure of MID. Additionally, for people with functional declines at 6 weeks, we established a secondary outcome (functional recovery), which was defined as returning to pre-hospitalization baseline function after an observed decline at 6 weeks.

Patients who completed all measurements were also classified into pre-defined functional trajectories based on changes in their functional dependence between their baseline and the 12-week follow-up. Three trajectories (A, B, and C) included patients whose ADL dependence score at week 12 was at least as good as at baseline, two trajectories (D and E) included patients whose dependence score was higher at week 12, and one trajectory (F) included patients whose dependence score was lower at week 12. Trajectory A included patients who had not declined between pre-hospitalization and week 6, and had no decline between weeks 6 and 12. Trajectory B included patients who declined between pre-hospitalization and week 6, but recovered to baseline levels of dependence at week 12. Trajectory C included patients who improved between pre-hospitalization and week 6, but declined to baseline levels of dependence at week 12. Trajectory D included patients who declined between pre-hospitalization and week 6 and who did not recovery to baseline levels of dependence at week 12. Trajectory E included patients who did not decline between pre-hospitalization and week 6, but declined between weeks 6 and 12. Finally, trajectory F included patients who improved between pre-hospitalization and week 6 and who did not decline to baseline levels of dependence at week 12.

Descriptive statistics were used to characterize the cohort at baseline and change rates. We used Pearson χ^2^ and independent t-test to examine differences in baseline characteristics with respect to whether or not the surrogates participated.

Univariate and multivariate logistic regression analyses were used to assess the possible factors associated with functional decline at week 12 after discharge. For these analyses, we dichotomized functional decline (yes/no), grouping those patients whose dependence score at week 12 was equal or higher than that at pre-hospitalization baseline into the negative category because we considered that both groups shared similar care needs. The univariate associations between each potential predictor were tested (with enter method) for significant associations with functional decline. Separate multivariate models were first fit for each of 3 domains (sociodemographic, illness and physiological factors, and hospitalization) by including as independent variables those of each domain that showed a statistically significant association (P< 0.10) in the univariate analyses. A final multivariate logistic regression logistic model was chosen by including the most strongly predictive factors from each of the individual models, as well as other significant factors at the 0.10 level. All multivariate models were produced using the forward step method. Goodness-of-fit and regression diagnostics for the models were assessed using methods described elsewhere [13].

Sample size calculation was based on the rule of thumb that 15 subjects per predictor are needed for a reliable equation [14]. We recruited a minimum of 90 participants assuming a maximum of 6 predictors. All analyses were performed with the SPSS statistical software program (SPSS version 19.0; IBM SPSS, Chicago, Illinois).

## Results

### Participants

Of the 107 hospitalized patients with COPD exacerbations, 4 were excluded because they had a length of stay longer than 30 days. Therefore, 103 patients were finally included.

Patients were generally older with moderate-to-severe COPD and 87 had a low risk of hospitalizations linked to AE, 22 of them had only one hospitalization. Of these, 55% had frailty and 32% reported having one or more ADL dependencies at baseline. Baseline characteristics are described in Table 1.

**Table 1:** Characteristics of the Study Population at Baseline. (N = 103).

Variables	Mean±SD or n (%)
**SOCIODEMOGRAPHIC**	
**Age (years)**	71.0±9.1
**Men**	96 (93.2)
**Education level**	
**Without studies**	48 (46.6)
**Primary studies**	37 (35.9)
**Secondary studies**	18 (17.5)
**ILLNESSES AND PHYSIOLOGICAL FACTORS**	
**FEV**_**1**_ **(% predicted)**	52.2±15.2
**Airflow limitation**	
**Mild**	3 (2.9)
**Moderate**	60 (58.3)
**Severe**	33 (32.0)
**Very severe**	7 (6.8)
**Dyspnea(mMRC score)**	2.6±0.9
**Frailty**	
**Not frail**	27 (26.2)
**Vulnerable**	19 (18.4)
**Frail**	57 (55.3)
**Number of drugs**	9.0±4.2
**Number of comorbidities**	
**0–2**	75 (72.8)
**≥3**	28 (27.2)
**Functional Comorbidity Index**	1.68±1.31
**HOSPITALIZATION CHARACTERISTICS**	
**Length of stay(days)**	11.3±7.4
**Hospitalizations in previous year**	
**0–1**	87 (84.5)
**≥2**	16 (15.5)
**OUTCOME MEASURES**	
**ADLs with dependence**	
**None**	70 (68.0)
**1**	11 (10.7)
**2**	10 (9.7)
**≥3**	12 (11.6)

**Abbreviations:** N: Number of cases that reported data; FEV1, forced expiratory volume in 1 second; mMRC, modified Medical Research Council.

At follow-up, 101 (98%) participated in the study 6 weeks after hospital discharge, and 100 (97%) participated at 12 weeks. All 3 patients lost to follow-up at the final time point had died. The percentage of interviews with surrogates increased from 0% in the hospital to 25.7% at the week 6 and 31% at the week 12 assessment after discharge. Thus, of the 103 participants in the sample, 34 (33%) were interviewed via surrogates at any follow-up time point. Most of the baseline characteristics of these patients were not significantly different from those who participated in all interviews; sex (P = 0.132), education level (P = 0.205), FEV_1_% (P = 0.137), severity of airflow limitation (P = 0.553), dyspnea (P = 0.096), frailty (P = 0.124), number of drugs (P = 0.693), number of comorbidities (P = 0.909), length of stay (P = 0.180), number of hospitalizations in the previous year (P = 0.458), and ADLs with dependence (P = 0.135). Nevertheless, the patients who participated in all interviews were younger, with a mean age of 69.7 ± 8.4 vs 73.9 ± 9.9 (P = 0.025).

### Rates and trajectories of change

**Fig 1:**
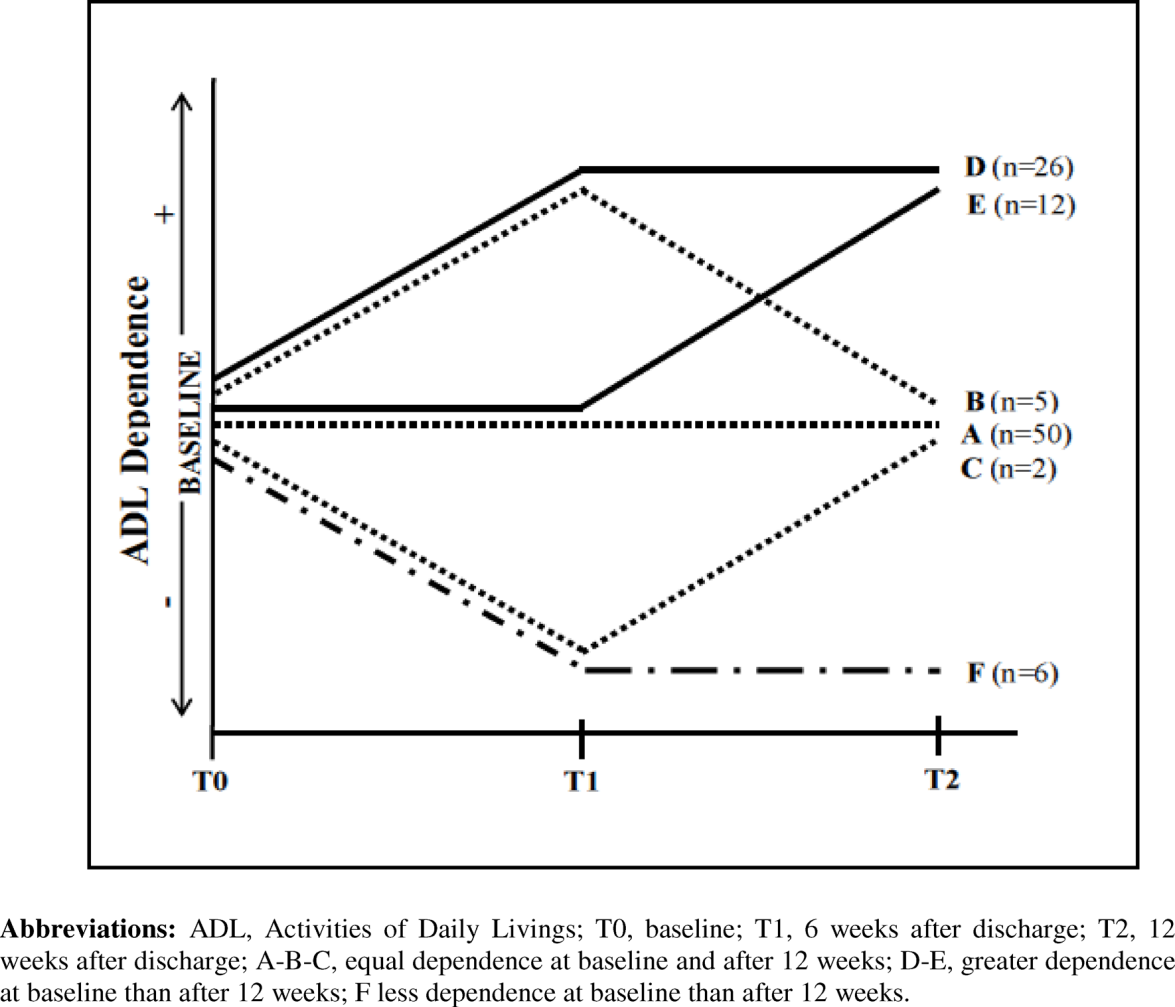
Trajectories of change between pre-hospitalization (T0) and 6 (T1) and 12 weeks (T2) after hospital discharge.

Of the 101 patients interviewed at week 6, 31 (30.7%) patients experienced decline, 62 (61.4%) had no change, and 8 (7.9%) had a pattern of improvement. Of those who declined, 5 (16.1%) achieved a full recovery to pre-hospitalization baseline function at week 12 after discharge. Of these who improved, 6 retained this improvement at week 12 and 2 returned to baseline function. “Fig 1” represents a diagram displaying each of the trajectories of change of those whose dependence score at week 12 were equal (trajectories A, B, and C), higher (trajectories D and E), or lower (trajectories F) compared with baseline (2 weeks before hospitalization). Of the 62 patients who experienced no change at 6 weeks, 50 (86.6%) kept their baseline function at week 12, but 12 (19.4%) declined between weeks 6 and 12.

At 12 weeks, as a consequence of the trajectories of change experienced, 38 (38%) patients showed a decline with respect to baseline function (the mean of ADL-score changed from 0.97 at baseline to 3.63 at week 12), 57 (57%) had not declined (50 had a trajectory of permanent no change), and 6 had a pattern of improvement (the mean of ADL-score changed from 3.17 to 1.17).

### Predictors of functional decline at week 12

Results of the univariate analyses are presented in Table 2. These unadjusted analyses provide a relative measure of the odds of experiencing functional decline at week 12 after hospital discharge in participants with specific characteristics compared with those without these characteristics. For example, the frailty subgroup was significant when using the not frail subgroup as the reference in the analysis. Based on these results, 5 variables were selected for multivariate analyses; age, dyspnea, frailty, length of stay, and number of hospitalizations.

**Table 2:** Univariable Logistic Regression Predicting Functional Decline at Week 12 after Hospital Discharge.

Variables	Odds Ratio (95% CI) Confidence intervaI)	P-value
**Sociodemographic**		
**Age (years)**	1.05 (1.00–1.10)	0.05
**Women**	0.63 (0.12–3.44)	0.60
**Education level**		
**Without studies**	Reference	
**Primary studies**	1.22 (0.50–2.98)	0.66
**High school or university**	0.85 (0.27–2.69)	0.79
**Illness and physiological factors**		
**FEV**_**1**_ **(% predicted)**	1.00 (0.97–1.03)	0.88
**Airflow limitation**		
**Mild**	Reference	
**Moderate**	1.08 (0.92–12.59)	0.95
**Severe**	1.33 (0.11–16.39)	0.82
**Very severe**	2.67 (0.16–45.14)	0.50
**Dyspnea (mMRC score)**	1.725 (1.08–2.77)	0.02
**Frailty**		
**Not frail**	Reference	
**Vulnerable**	3.35 (0.82–13.78)	0.09
**Frail**	5.75 (1.75–18.86)	0.004 18.87)
**Number of comorbidities ≥3**	1.03 (0.41–2.57)	0.96
**Hospitalization characteristics**		
**Length of stay (days)**	1.12 (1.04–1.21)	0.004
**Hospitalizations in previous year ≥2**	3.33 (1.10–10.11)	0.03

**Abbreviations:** FEV1, forced expiratory volume in 1 second; mMRC, modified Medical Research

Table 3 shows the results of multivariate models of predictors of functional decline. Model 1 suggests that dyspnea and frailty are two physiological factors associated with higher odds of decline at week 12. Model 2 suggest that length of stay and age are also associated with higher odds of functional decline. In the final model, only frailty, dyspnea, and length stay were retained. According this model, it is expected that the odds of undergoing a functional decline between 2 weeks before hospitalization and 12 weeks after discharge increases almost 400% for patients with frailty (odds ratio [OR] = 3.97; 95%CI 1.13–13.92), 85% for every 1-unit increase on the mMRC dyspnea scale (OR = 1.85;95%CI1.05–3.26), and 12% for every 1-day increase in the length of hospital stay (OR = 1.12;95%CI1.03–1.22).

**Table 3:** Predictors of Functional Decline at Week 12 after Hospital Discharge.

Variables	Odds Ratio (95% confidence interval)		
	Model 1	Model 2	Final model
**Dyspnea**	1.69 (1.00–2.851)^a^	-	1.85 (1.05–3.26)^a^
**Frailty**			
**Not frail**	Reference	-	Reference
**Vulnerable**	4.23 (0.96–18.53)^b^		2.30 (0.46–11.54)
**Frailty**	5.23 (1.56–17.57)^a^		3.97 (1.13–13.92)^a^
**Length of stay**	-	1.11 (1.03–1.20)^a^	1.12 (1.03–1.22)^a^
**Hospitalizations in year previous year**	-	3.05 (0.89–10.49)^b^	Excluded
**Age (years)**	Excluded	1.06 (1.00–1.12)^a^	Excluded
**Adjusted R**^**2**^	18.3%	24.4%	29.8%

Superscripts

^a^ P < 0.05

^b^ P < 0.10

## Discussion

To our knowledge, this is the first study to assess the pre-hospitalization functional status of patients with AE-COPD to evaluate the impact of the patients’ hospitalization on the development of disability in the middle term. Prospective development of disability after hospitalization for AE-COPD was a common occurrence in our cohort. More than one third of patients hospitalized for AE-COPD showed new or additional dependence in ADLs after 3 months, and similar rates of functional decline were seen at 6 weeks. The finding of 6 different trajectories of functional change in the sample of 101 patients who were assessed 3 times illustrates the inter-individual heterogeneity possible in the functional course after hospital discharge.

Length of stay, dyspnea, and frailty were independent predictors of functional decline at 3 month following hospitalization. Our findings suggest that disability progression after hospitalization for AE-COPD may be driven by the hospitalization experience itself as well as mobility limitations due to breathlessness and frailty. Nevertheless, length of stay could also represent both hospitalization experience and severity of illness [15]. Dyspnea has also been identified as a potential risk factor for disability progression over time in non-hospitalized COPD patients [3]. Frail patients in our sample were strikingly vulnerable to new or additional dependence in ADLs. Frailty was previously shown to increase the risk of disability progression following hospitalization in older adults [4, 16]. Although the literature recognizes that vulnerable older people are at the highest risk of developing dependence in ADLs [16], our results show that age was not a determinant of decline for AE-COPD. The initial association between age and functional decline diminished when it was adjusted for illness and physiological factors, as also seen in previous studies using other populations [4]. The lack of association with some risk factors previously identified in the literature (such as number of comorbidities) merits further investigation in other studies.

Our study suggests that the process of recovery after decline is unlikely in the short term. Only one sixth of patients who experienced functional decline at the first time interval recovered during the next time interval. The natural history of functional recovery after functional decline has not been previously described for adults hospitalized for AE-COPD, and we have insufficient information to contrast our findings in this population with other studies. Nevertheless, our recovery rates are lower than those reported over shorter time intervals in previous studies in hospitalized older adults [17].

The results of the current study suggest that adults hospitalized for AE-COPD may have dramatically higher functional care needs after than before their hospitalization. Consequently, the demands on caregivers will markedly increase. These results have important implications for healthcare delivery during as well as after hospitalization. Preventing functional decline in frail hospitalized patients might be the key to improving patient outcomes. Some simple interventions like a combination of balance and strength training have shown encouraging benefits for preventing functional decline in older patients [18,19].

### Strengths and limitations of study

The particular strengths of this study are the use of a pre-hospitalization assessment and two additional measurement time points following discharge, which allowed an assessment of patients’ levels of dependence before hospitalization and the functional trajectories that occurred between the observation points.

Our study has several limitations. Most of the data on dependence were collected directly from the patient, but some information was reported by surrogates. This approach has drawbacks because reported disability relies, at least partially, on the subject who reports the events perception of them. However, some studies have shown that patients and proxies provide reliable interrater information when they reported disability during interviews [20]. Further supporting evidence for the validity of our data is that patients who participated in all the interviews had similar characteristics to others who did not. Second, although measurements were taken at 3 time points to determine the time course of functional change after hospitalization, it is likely that there were additional functional transitions that occurred between these time points [21]. Third, trajectories over time may be influenced by continued health service use and other environmental factors. All patients were recruited from an urban hospital and data were lacking on post-acute rehabilitation services. Nevertheless, we know that rehabilitation services were not widely used by the patients. Fourth, the patterns of recovery after functional decline were only studied over a short term (between weeks 6 and 12). The duration of recovery period may be longer [4]; consequently, our results are likely to underestimate recovery rates. Fifth, the final model explained only 29.8% of the variance. Although being acceptable, it may be speculated that some other factors not included in our model could even further improve its predictive power (e.g. daily physical activities level). Finally, because of the small number of women in the cohort, the results should be cautiously generalized to women. Future prospective longitudinal studies are needed that analyze longer periods of follow-up with a more representative sample, including more females and patients from both rural and urban health facilities.

In conclusion, more than one third of the patients hospitalized for AE-COPD declined during the subsequent 12 weeks after discharge, with most of the decline occurring by week 6. These results have implications for hospital physicians and rehabilitation services, and efforts should be prioritized to rehabilitate frail patients. Studies are needed to determine interventions that would maximize the prevention of functional decline. For patients who decline and do not recover, social services should be also applied. Additionally, more studies are necessary to determine the relationship between disability progression and other outcomes often measured for adults hospitalized for AE-COPD (mortality, readmission rates, lung function measurements, and quality of life or patient satisfaction).
